# *Brucella suis* Seroprevalence and Associated Risk Factors in Dogs in Eastern Australia, 2016 to 2019

**DOI:** 10.3389/fvets.2021.727641

**Published:** 2021-09-21

**Authors:** Catherine C. Kneipp, Kate Sawford, Kate Wingett, Richard Malik, Mark A. Stevenson, Siobhan M. Mor, Anke K. Wiethoelter

**Affiliations:** ^1^Faculty of Veterinary and Agricultural Sciences, University of Melbourne, Melbourne, VIC, Australia; ^2^Kate Sawford Epidemiological Consulting Pty Ltd, Sydney, NSW, Australia; ^3^Local Land Services, Braidwood, NSW, Australia; ^4^New South Wales Department of Primary Industries, Orange, NSW, Australia; ^5^Centre for Veterinary Education, University of Sydney, Sydney, NSW, Australia; ^6^School of Animal and Veterinary Sciences, Charles Sturt University, Wagga Wagga, NSW, Australia; ^7^Institute of Infection, Veterinary and Ecological Sciences, University of Liverpool, Liverpool, United Kingdom

**Keywords:** *Brucella suis*, hunting dogs, Australia, feral pigs, zoonosis

## Abstract

*Brucella suis* is a zoonotic disease of feral pigs that also affects pig hunting dogs, pig hunters, veterinarians and veterinary staff. In recent years the incidence of *B. suis* in the eastern Australian states of New South Wales (NSW) and Queensland (QLD) has increased. A cross-sectional study was conducted to document the seroprevalence, geographical extent and risk factors for *B. suis* in dogs at-risk of contracting the disease. Eligible dogs were those that were known to hunt or consume feral pig meat. Dogs were enrolled through private veterinary clinics and/or directly by District Veterinarians in six regions of NSW and QLD. Blood was collected by venepuncture and tested for *B. suis* antibodies using the Rose Bengal Test (RBT) followed by a Complement Fixation Test (CFT) if they returned a positive RBT. Owners were invited to complete a questionnaire on the dogs' signalment, husbandry including hunting practices and locations, and any clinical signs referable to brucellosis. Of the 317 dogs included in the prevalence survey, 21 were seropositive returning a survey-adjusted true seroprevalence of 9.3 (95% CI 0.45 to 18) *B. suis* positive dogs per 100 dogs at-risk. True seroprevalence ranged from 0 to 24 *B. suis* positive dogs per 100 across eastern Australia, with the highest prevalence in central west NSW and southern QLD. Adjusted for other factors, dogs that shared a household with other seropositive dogs and those that traveled away from their home regions to hunt were more likely to be seropositive. Clinical signs at presentation were not predictive of serostatus, with seropositive and seronegative dogs equally likely to present with signs consistent with brucellosis. The results obtained from this study show that *B. suis* exposure is relatively common in dogs that have contact with feral pigs, with one in 10 testing seropositive. Further studies are needed to understand the progression and risk of transmission from seropositive dogs.

## Introduction

Brucellosis is a bacterial zoonotic disease caused by members of the genus *Brucella*. These bacteria are gram-negative facultative intracellular coccobacilli of the class *Alphaproteobacteria. Brucella* spp. localize in lymphoreticular tissue and primarily cause disease in the reproductive tissues in their natural hosts (1). Transmission between hosts occurs by contact with infected body fluids and tissues via mucous membranes or broken skin, as well as through ingestion and inhalation. The infection can range from mild to severe multiorgan disease and from subclinical to acute or chronic presentations. Brucellosis is considered one of the most common zoonotic diseases worldwide, with approximately 500,000 people diagnosed annually (2, 3). Humans are mostly infected following exposure to the bacteria from domestic or wild animals or animal derived products (4). Of the five *Brucella* spp. known to cause disease in humans, namely *B. melitensis, B. abortus, B. suis, B. canis* and *B. ceti* (5, 6), only *B. suis* is present in Australia. *Brucella suis* has five biovars, with swine being the natural host for biovars 1, 2 and 3 (7, 8). *Brucella suis* biovars 1 and 3 are found worldwide with biovar 2 found predominantly in Europe in wild boar. Venereal infection is considered a common form of transmission in pigs as is the consumption of birth products such as fetal tissue, membranes and fluids or feed and water contaminated by such products (9).

First diagnosed in domestic pigs in the state of Queensland (QLD) in 1936 (10), *B. suis* biovar 1 was a major cause of production loss in commercial piggeries until a test and slaughter program eradicated it from domestic pigs in 1968. However, the presence of *B. suis* biovar 1 has been detected in feral pigs (*Su*s *scrofa*) in QLD since 1976 (10), and more recently it has been detected in feral pigs in NSW (11). To date *B. suis* has not been reported in feral pigs in any other Australian states or territories (12–14). *Brucella suis* biovar 1 is highly pathogenic to humans, second only to *B. melitensis* (6, 15) in terms of virulence and is capable of causing serious and chronic systemic disease (4–6, 16). Although a rare infection, *B. suis* is the only cause of domestically acquired brucellosis in humans in Australia with recreational and occupational exposure to feral pigs the most common risk factor for infection (16, 17).

Between 100,000 and 200,000 people are estimated to hunt feral pigs in Australia each year, with the majority of hunters using domestic dogs to locate and capture pigs (18, 19). Dogs are used to track, find and chase (“flush out”) pigs; to stop them by chasing until a pig is cornered or exhausted and therefore remains stationary without direct contact (“bailing”); or holding (“lugging”) where the dog bites the pig, often on the ears and head but also the extremities, until the hunter either shoots the pig at close range or kills it by stabbing it in the heart with a knife. This level of contact and exposure to the natural host of *B. suis* is believed to increase the risk of infection in pig hunting dogs. Indeed, between 2011 and 2015 there was a marked increase in the number of dogs presented to veterinary clinics in NSW with signs consistent with brucellosis (20). A subsequent investigation by Mor et al. (21) concluded that brucellosis was an emerging infectious disease in dogs involved in feral pig hunting in Australia and that infected dogs posed a potential zoonotic risk to humans in close contact.

*B. suis* infection in dogs following exposure to feral pigs through hunting or other contact has been reported worldwide (22–29) and although the exposure of hunting dogs to different zoonotic pathogens has been documented in Europe and Central America (30, 31) no studies have systematically investigated *B. suis* infection in dogs. Therefore, the primary objectives of this study were two-fold; firstly, to estimate the seroprevalence of *B. suis* among at-risk dogs in eastern Australia and secondly to identify characteristics and circumstances of dogs that increased their risk of being seropositive. A further objective was to characterize the demographics and husbandry of the pig hunting dog population. For the purpose of this study at-risk dogs comprised those with a known history of pig hunting or dogs who routinely consumed feral pig meat or had access to feral pig carcasses and pig hunting equipment. As exposure to feral pigs is already known to be a risk factor for *B. suis* this study has focussed on more nuanced aspects related to specific hunting practices and other household exposures that may contribute to infection in this hard-to-reach population.

## Materials and Methods

### Study Population and Owner Questionnaire

This was a cross-sectional study carried out in south west QLD and NSW between December 2016 and December 2019. The source population comprised domestic dogs that were resident in five Local Land Services (LLS) regions in NSW namely the North West, Central West, Hunter, Central Tablelands and South East, as well as one Local Government Area (LGA) in south west QLD, the Goondiwindi Region. These regions were selected on the basis of their geographical location, the presence of feral pigs and the level of interest and co-operation from the respective LLS District Veterinarians and private veterinarians.

Private veterinary clinics in each of the selected regions were invited by the District Veterinarians and researchers to participate in the study. Following agreement to take part the veterinary clinics were provided with equipment and instructions for recruitment, administration of the study questionnaire and blood sample collection. Dogs were enrolled by both the private veterinary clinics and/or by District Veterinarians in each of the study areas. Only one dog per owner was eligible to be included in the study. Following consent to take part dog owners were asked to complete a questionnaire to provide details of their dog's age, breed, sex and reproductive status (entire vs. neutered), origin, husbandry, diet and recent health status as well as details of their hunting history including hunting regions and routine hunting practices. The questionnaire was comprised of short open-answered questions as well as single and multiple selection closed-ended questions. Incomplete questionnaires were accepted. All questionnaires were assigned a unique identifier with regional codes; the owner's details were removed to preserve confidentiality. Owner notification of results, management and follow-up of all positive and inconclusive cases was carried out by the submitting veterinarian.

### Blood Sample Collection and Laboratory Analysis

At the time of enrolment into the study a blood sample was collected from each study participant by venepuncture. Clotted whole blood or serum was submitted to the NSW State Veterinary Diagnostic Laboratory at the Elizabeth MacArthur Agricultural Institute (EMAI) for *B. suis* antibody testing.

The Rose Bengal Test (RBT) was used as an initial screening test. The RBT is a rapid slide agglutination test, designed to detect IgG and IgM antibodies to smooth *Brucella* spp. (including *B. suis*) with a high degree of sensitivity (32, 33). The RBT, as performed at EMAI, has a diagnostic sensitivity of between 81% and 87% and a diagnostic specificity of 86.3% (34). Sample serum and a standardized suspension of stained *B. abortus* antigen were mixed in equal volumes in an acidified buffered medium of pH 3.65 ± 0.05. Any visible agglutination caused by the formation of antigen-antibody complexes within four minutes was considered a positive result. Results were reported as negative, 1+ low positive, 2+ medium positive, 3+ high positive based on the degree of agglutination (32).

The Complement Fixation Test (CFT) was used as a confirmatory test consistent with standard practice (4, 35, 36). A modified version of the standard CFT was used to decrease the occurrence of anti-complementary (AC) reactions. Specifically, the standard incubation of 60 ± 2 °C for 45 ± 5 min incubation was replaced with an incubation of 53 °C for 30 min (37). The CFT has a diagnostic sensitivity of 54% and diagnostic specificity of between 95 and 99% (37). Results were reported as reciprocal titer measurements ranging from 4 (a low seropositive result) to 128 (a high seropositive result), with EMAI reporting CFT titres of <4 as negative, CFT titres of 4–8 as inconclusive and CFT titres of ≥16 as positive.

Consistent with standard practices at EMAI, seropositive cases were defined as those with a positive RBT result and a CFT titer of ≥16, while seronegative animals were defined by a negative RBT result (series interpretation). According to EMAI, dogs with a positive RBT and an anti-complementary CFT or a CFT titer ≤ 8 should be retested in six weeks. It was beyond the scope of the survey to recall dogs for retesting and as such these cases were classified as inconclusive and excluded from further analyses in this study.

### Statistical Analyses

Based on an assumed seroprevalence of 3%, a combined sensitivity of 45% and specificity of 100% for the RBT and CFT tests (series interpretation) and to achieve 95% confidence that our estimate of *B. suis* exposure was within 5% of the true population value, a total of 102 dogs from each region needed to be enrolled to meet the requirements of the study (38).

The apparent *B. suis* seroprevalence in each region and its associated 95% confidence intervals were calculated using EpiTools (38, 39). Apparent seroprevalence estimates were then expressed as true prevalence estimates to account for imperfect diagnostic test sensitivity using the Rogan-Gladen estimator (40). Survey design adjustment procedures (41) were then used to provide a summary estimate of seroprevalence, accounting for differences in sample numbers across the six study regions. For our survey adjustment we assumed the ratio of dogs to humans would be approximately equal across the six study areas. Each dog that took part in the study was assigned a weight equal to human population size (42) divided by the corresponding number of study dogs in each region. The survey adjusted apparent and true seroprevalence of *B. suis* across all regions was then calculated as a weighted average of the region estimates.

Questionnaire data and test results were uploaded to the University of Sydney's Research Electronic Data Capture (REDCap) server and subsequently cleaned and analyzed in IBM SPSS Statistics version 26 and R version 4.0.3 using the contributed “ggplot2” (43), “scatterpie” (44) and “ggsn” packages (45). The age of each dog in months was re-classified into age groups (0 to 18 months, 19 to 60 months, 61 to 108 months and 109 to 156 months) and breed categorized according to standard classifications based on size (small, medium and large). Each dog was categorized into “north” or “south” location based on their sampling location postcode with the north to south sampling location extent measured in kilometers, as the crow flies, and divided into halves to demarcate the two categories. On the questionnaire, owners were able to list up to three locations where they took the dog hunting. The locations were recorded as state, postcode and/or nearest town/shire, in a free text option. Hunting postcodes, towns and shires were checked to identify if they were located in the hunter's home region (based on LLS region/LGA); dogs that hunted in at least one area outside the hunter's home region were classified as hunting “away” whereas those that hunted exclusively within the home region were classified as hunting “home”. Postcodes were used to map hunting locations. Digital maps for state, LGA and postcode boundaries were obtained from the Australian Bureau for Statistics (46) and LLS region boundaries from the NSW State Government (47).

Descriptive statistics were calculated for each exposure variable stratified by sex to investigate the influence of sex on *B. suis* seropositivity and husbandry practices/exposures. All variables that were deemed to be of adequate quality (missing values <12%) were selected for further analysis. A series of univariable logistic regression models were used to identify putative risk factors for *B. suis* seropositivity. For variables that contained zero cell frequencies the odds ratio (OR) and 95% confidence interval (CI) were calculated using the Haldane-Anscombe correction in OpenEpi version 3.01 (48–50). Exposure variables with an unconditional association with seropositivity that had a *p*-value ≤ 0.25 were selected as candidate explanatory variables for logistic regression modeling. Candidate explanatory variables were checked for collinearity and confirmed to have a low variance inflation factor (VIF <3) before fitting the model. Candidate explanatory variables were selected for inclusion in the logistic regression model based on backward stepwise elimination (51) and least angle selection and shrinkage operator (LASSO) penalties (52). The same candidate explanatory variables were identified using both methods. Plausible two-way interactions between each of the explanatory variables retained in the final model were tested and none were significant at *p*-value ≤ 0.05. A Receiver Operating Characteristic (ROC) curve was constructed based on the seropositive status of dogs as predicted by the model. The area under the ROC curve was used to measure of the model's ability to predict seropositivity.

## Results

### Sample Population

Blood samples from 384 dogs were collected across the six study regions. Of these 55 were excluded from the analyses for the following reasons: same household as a dog that was already enrolled (*n* = 33); sample unsuitable for serology testing (*n* = 17); and did not meet the inclusion criteria of “at-risk” (*n* = 5). A further 12 dogs returned inconclusive serology results and were excluded from further analyses. Therefore, 317 at-risk dogs were included in the study. Owner questionnaires were available for 274 dogs due to varying levels of completion.

### Signalment, Clinical Presentation and Husbandry Practices of At-Risk Dogs

Signalment and clinical presentation of dogs included in the study are shown in Table 1. Entire male dogs were predominant in the study group. The median age was 54 months (4.5 years) with a range of 1.5 to 180 months. The most common dog type or breed reported was the Bull Arab (15%), which was initially a cross between English Bull Terriers, Greyhounds and Pointers (53, 54) and developed in Australia in the 1970s specifically for hunting feral pigs. However, when accounting for crossbreeds, Bull Arabs crossed with other large breeds such as Bullmastiffs, Wolfhounds and Great Danes represented 47% of the study group. Medium sized dogs (10%) comprised mainly working breeds such as kelpies, Australian cattle dogs, Border collies and their crosses. The two small dogs included were Jack Russell terriers; one was taken hunting and both had access to pig carcasses. In the week prior to being tested, 75 of 271 (28%) of dogs had clinical signs that could be attributed to brucellosis. Of these, lethargy, lameness/joint signs and orchitis were most common. Of 93 females for which information was available, 10% had aborted a litter of pups at some stage in their lives.

**Table 1:** Signalment and clinical presentation of dogs at-risk of *Brucella suis* in eastern Australia, 2016 to 2019.

**Variable**	**Variable categories**	**N**	**%**
*Signalment*			
Sex (*n* = 273)	Male	167	61.2
	Female	106	38.8
Sterilization status (*n =* 273)	Entire	223	81.7
	Desexed	50	18.3
Age in months (*n =* 271)	0–18	35	12.9
	19–60	128	47.2
	61–108	93	34.3
	109–180	15	5.5
Breed (*n =* 272)	Small	2	0.7
	Medium	28	10.3
	Large	242	89
*Clinical presentation*			
Presence of clinical signs in the	Yes	75	27.7
last week (*n* = 271)	No	196	72.3
In dogs with signs, type of clinical signs:			
Lethargy (*n* = 73)	Yes	37	50.7
	No	34	46.6
	Don't know	2	2.7
Anorexia (*n =* 71)	Yes	23	32.4
	No	46	64.8
	Don't know	2	2.8
Fever (*n =* 73)	Yes	19	26
	No	46	63
	Don't know	8	11
Lameness (*n =* 71)	Yes	36	50.7
	No	31	43.7
	Don't know	4	5.6
Joint signs (*n =* 70)	Yes	26	37.1
	No	38	54.3
	Don't know	6	8.6
Back pain (*n =* 67)	Yes	11	16.4
	No	48	71.6
	Don't know	8	11.9
Ocular signs (*n =* 67)	Yes	5	7.5
	No	55	82
	Don't know	7	10.4
Orchitis (*n =* 48)	Yes	17	35.4
	No	30	62.5
	Don't know	1	2.1
Ever aborted (*n =* 93)	Yes	9	9.7
	No	79	84.9
	Don't know	5	5.4

*Questionnaires were partially or fully completed by 274 owners. Where incomplete data were recorded, the denominator used in the percentage calculation is indicated in parentheses after the variable name*.

**Figure 1:**
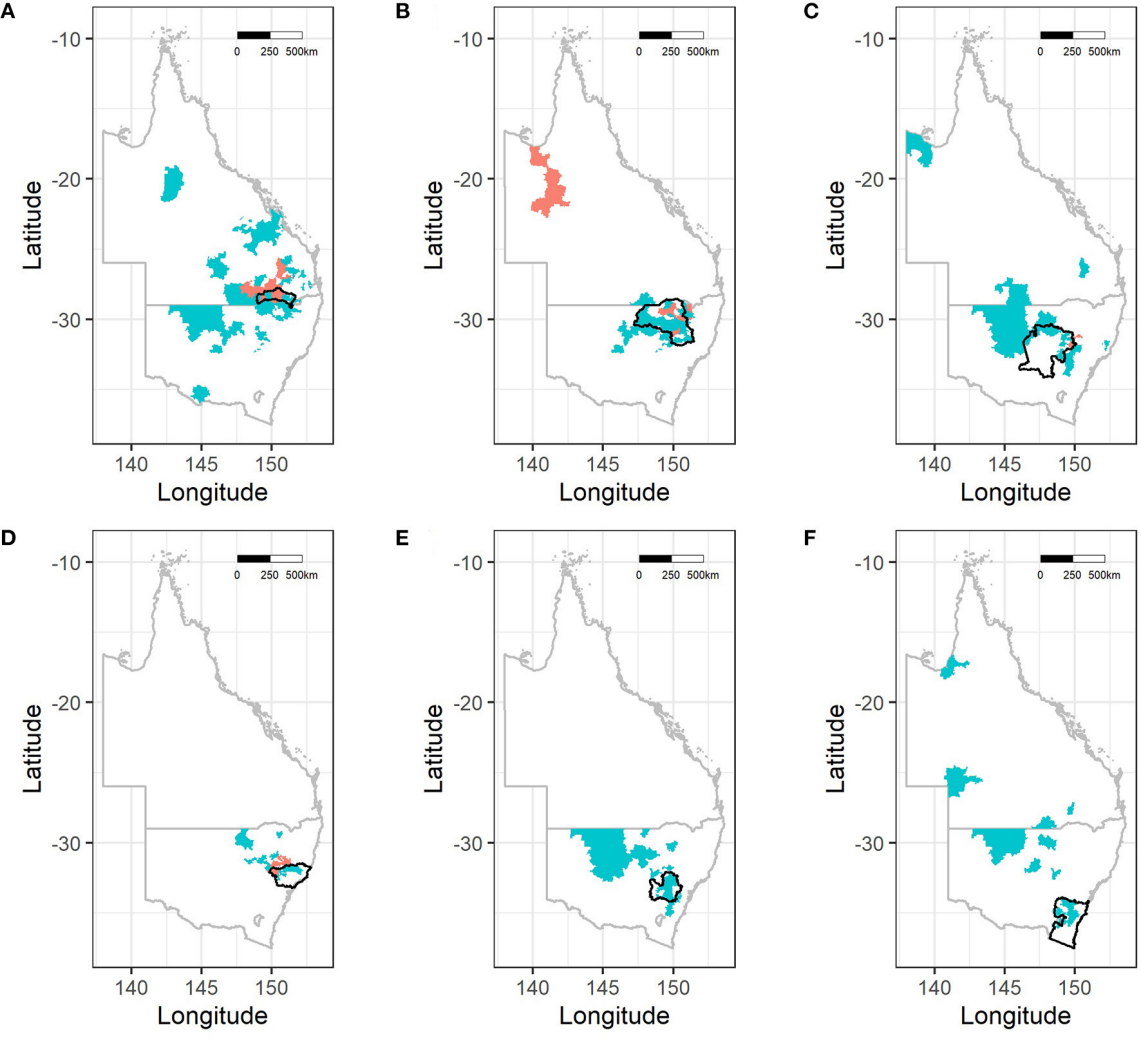
Postcode areas frequented by dogs for hunting based on Local Land Services (LLS) region or Local Government Area (LGA) from which dogs originated. **(A)** Goondiwindi Region, **(B)** North West, **(C)** Central West, **(D)** Hunter, **(E)** Central Tablelands, and **(F)** South East. Note that number of dogs sampled per region and sizes of postcode areas varies. Postcodes shaded in coral represent areas where seropositive dogs hunted. Since dogs hunted in multiple areas that does not infer location where transmission/infection has occurred.

Husbandry and hunting practices of dogs are shown in Table 2. Most of the dogs lived in households with other at-risk dogs (78%). Nearly all dogs (96%) were involved in hunting feral pigs, the majority of which (75%) hunted more than 12 times a year. Although the majority of dogs (61%) hunted exclusively in their home region, others left their home region to hunt, with some dogs traveling up to 2,000 km (Figure 1). Most of the dogs had close contact with feral pigs when hunting with 89% of dogs involved in the high contact practice of holding and lugging. Just over half of the dogs were fed pig meat (56%) and less than half of the dogs (42%) were given access to the carcass during or after the hunt. Meat and bones (specifically legs) were the preferred cuts fed with only 3% (4 of 151) of dogs fed offal from feral pigs.

**Table 2:** Husbandry and hunting practices of dogs at-risk of *Brucella suis* in eastern Australia, 2016 to 2019.

**Variable**	**Categories**	**N**	**%**
*Hunting practices*			
Ever hunted (*n* = 272)	Yes	260	95.6
	No	12	4.6
In dogs that hunt:			
Hunting frequency (*n* = 257)	<1/year	16	6.2
	1–6x/year	40	15.6
	7–12x/year	9	3.5
	>12x/year	192	74.7
Hunting location^†^ (*n* = 254)	Away	98	38.6
	Home	156	61.4
Hunting method:			
Finding/pointing (*n* = 260)	Yes	160	61.5
	No	100	38.5
Flushing/bailing (*n* = 260)	Yes	89	34.2
	No	171	65.8
Holding/lugging (*n* = 260)	Yes	231	88.8
	No	29	11.2
*Exposure to feral pig meat/carcass*			
Eats feral pig meat	Yes	151	55.9
(*n* = 270)	No	119	44.1
In dogs that eat feral pig meat,	<1/year	32	22.1
frequency of consumption	1–6x/year	43	29.7
(*n* = 145)	7–12x/year	13	9
	>12x/year	57	39.3
Other access to feral pig	Yes	108	41.9
carcasses (*n* = 258)	No	150	58.1
*Exposure to other at-risk dogs*			
Multiple hunting dogs in the	Yes	209	77.7
household (*n* = 269)	No	59	21.9
	Don't know	1	0.4
Mated to a dog that	Yes	86	32.1
hunts (*n* = 268)	No	172	64.2
	Don't know	10	3.7
Mated to a dog that eats feral pig	Yes	52	20.1
meat (*n* = 259)	No	189	73
	Don't know	18	6.9
Lives with dogs that eat feral pig	Yes	118	45.7
meat (*n* = 258)	No	138	53.5
	Don't know	2	0.8
Lives with *B. suis* seropositive	Yes	16	6.1
dog (*n* = 262)	No	197	75.2
	Don't know	49	18.7
Region (*n* = 274)	North	217	79.2
	South	57	20.8

*Questionnaires were partially or fully completed by 274 owners. Where incomplete data were recorded, the denominator used in the percentage calculation is indicated in parentheses after the variable name*.

† *Dogs which hunted in at least one area outside the hunter's home region were classified as hunting “away” whereas those which hunted exclusively within the home region were classified as hunting “home”*.

### Prevalence of *B. suis* in At-Risk Dogs

**Figure 2:**
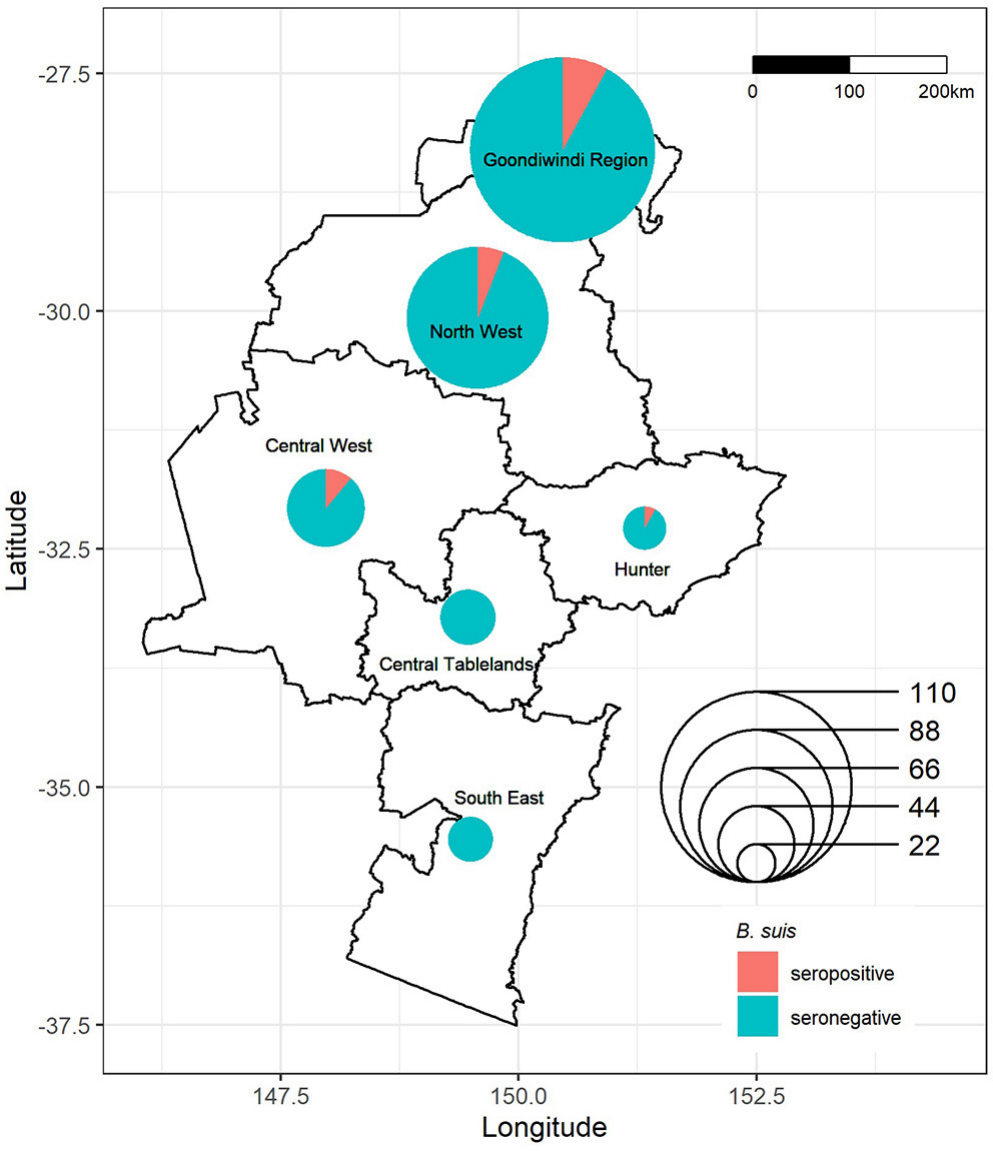
Location and *Brucella suis* serostatus of dogs sampled in eastern Australia, 2016–2019. Symbols map to the centroid of the Local Land Services (LLS) region or Local Government Area (LGA) from which dogs originated, with size corresponding to the number of samples obtained and color to the serostatus of dogs from that region.

Of 317 at-risk dogs included in the serosurvey, 21 dogs tested seropositive for *B. suis*, equivalent to an apparent prevalence of 6.6 (95% CI 4.1 to 10) positive dogs per 100 dogs at-risk (Table 3). Accounting for the imperfect sensitivity of the tests and differential sampling of dogs across the regions the survey adjusted true prevalence was 9.3 (95% CI 0.45 to 18) *B. suis* positive dogs per 100 dogs at-risk. Seropositive animals were detected in four of the six regions (Table 3, Figure 2). The true seroprevalence of *B. suis* by region ranged from 0 to 24 positive dogs per 100 dogs at-risk across the six regions, however the 95% confidence intervals for the regional seroprevalence estimates overlapped each other indicating that either seroprevalence did not vary by region or we had insufficient data to detect differences in regional seroprevalences if they did, in fact, exist.

**Table 3:** Apparent seroprevalence (AP) and true seroprevalence (TP) of *Brucella suis* in dogs in eastern Australia, by region, 2016 to 2019.

**Region**	**Number of**		**AP (95% CI)^†^**	**TP (95% CI)^†^**
	**Positive dogs**	**Negative dogs**		
Goondiwindi Region	9	98	8.4 (3.9–15.4)	17.8 (11.0–26.3)
North West	5	77	6.1 (2.0–13.7)	12.2 (6.0–21.3)
Central West	5	40	11.1 (3.7–24.1)	24.4 (12.9–39.5)
Hunter	2	23	8.0 (1.0–26.0)	16.0 (4.5–36.1)
Central Tablelands	0	32	0 (0–10.9)	0 (0–10.9)
South East	0	26	0 (0–13.2)	0 (0–13.2)
Total	21	296	6.6 (4.1–9.9)	14 (10–18)
Survey adjusted prevalence	-	-	4.6 (0.18–8.9)	9.3 (0.45–18)

† *Number of B. suis positive dogs per 100 dogs at-risk*.

### Risk Factors for *B. suis* in At-Risk Dogs

Unconditional associations between each of the hypothesized explanatory variables and *B. suis* seropositivity are shown in Table 4. Male dogs were nearly four times more likely to test positive than female dogs (10.2% compared to 2.8%; OR 3.9; 95% CI 1.1 to 14; *p* = 0.016). There was no association between seropositivity and de-sexing status or age, even when stratified by sex (data not shown). Holding and lugging was associated with seropositivity (OR 5.1; 95% CI 0.30 to 87; *p* = 0.035) while consumption of pig meat (OR 0.77; 95% CI 0.31 to 1.9; *p* = 0.58) and access to feral pig carcasses (OR 0.88 95% CI 0.33 to 2.3; *p* = 0.79) did not increase the odds of seropositivity. Dogs that lived in the north region (OR 12; 95% CI 0.71 to 200; *p* = 0.002) and those that traveled outside their LLS region/LGA for hunting were significantly more likely to be seropositive (OR 2.7; 95% CI 1.0 to 7.2; *p* = 0.045). One hundred hunting postcodes were identified in the survey with 19 of them being accessed by seropositive dogs (Figure 1). The strongest association was between seropositivity and dogs that shared a household with *B. suis* positive dogs (OR 16; 95% CI 4.9 to 54; *p* < 0.001). All seropositive dogs shared a combination of feeding, watering or sleeping equipment with the other dogs in the household. Despite clinical signs consistent with brucellosis being common in the study population, only 8% of clinically conspicuous dogs were seropositive and none of the clinical presentations were statistically significantly associated with seropositivity (Table 5).

**Table 4:** Unconditional associations between *Brucella suis* seropositivity and signalment, clinical presentation and husbandry practices.

**Variable**	**Categories**	**Number (%) of**		**OR (95% CI)**	***p*** -value^*^
		**Positive dogs**	**Negative dogs**		
Total number of dogs		20(7.3)	254(92.7)		
*Signalment*					
Sex (*n* = 20 | 253)	Male	17(85)	150(59.2)	3.89 (1.12–13.62)	0.016
	Female	3(15)	103(40.7)	Reference	
Sterilization status (*n* = 20 | 253)	Entire	17(85)	206(81.4)	1.29 (0.36–4.59)	0.684
	Desexed	3(15%)	47(18.6)	Reference	
Age in months (*n* = 20 | 251)	0–18	2(10%)	33(13.1)	Reference	0.437
	19–60	11(55%)	117(46.6)	1.55 (0.33–7.35)	
	61–108	7(35%)	86(34.3)	1.34 (0.27–6.8)	
	109–180	0(0)	15(6)	2.31 (0.10–51.11)^†^	
Breed (*n* =20 | 252)	Small	0(0)	2(1)	0.41 (0.02–8.90)^†^	0.856
	Medium	2(10)	26(10.3)	0.96 (0.21–4.36)	
	Large	18(90)	224(88.9)	Reference	
*Clinical presentation*					
Presence of clinical signs in last	Yes	6(30)	69(27.5)	1.13 (0.42–3.06)	0.811
week (*n* = 20 | 251)	No	14(70)	182(72.5)	Reference	
*Hunting practices*					
Ever hunted (*n* = 20 | 252)	Yes	18(90)	242(96)	0.37 (0.08–1.83)	0.268
	No	2(10)	10(4)	Reference	
In dogs that hunt:					
Hunting frequency (*n* = 18 | 239)	<1/year	0(0)	16(7.1)	2.68 (0.15–46.99)^†^	0.246
	1–6x/year	4(22.2)	36(15.1)	1.41 (0.44–4.54)	
	7–12x/year	0(0)	9(3.8)	1.54 (0.08–27.87)^†^	
	>12x/year	14(77.8)	178(74.5)	Reference	
Hunting location (*n* = 18 | 236)	Away	11(61.1)	87(36.9)	2.69 (1.01–7.2)	0.045
	Home	7(38.9)	149(63.1)	Reference	
Hunting method:					
Finding/pointing	Yes	12(66.7)	148(61.2)	1.27 (0.46–3.5)	0.64
(*n* = 18 | 242)	No	6(33.3)	94(38.8)	Reference	
Flushing/bailing	Yes	7(38.9)	82(33.9)	1.24 (0.46–3.32)	0.669
(*n* = 18 | 242)	No	11(61.1)	160(66.1)	Reference	
Holding/lugging	Yes	18(100)	213(88)	5.11 (0.30–87.08)^†^	0.035
(*n* = 18 | 242)	No	0(0)	29(12)	Reference	
*Exposure to feral pig meat/carcass*					
Eats feral pig meat (*n* = 20 | 250)	Yes	10(50)	141(56.4)	0.77 (0.31–1.92)	0.58
	No	10(50)	109(43.6)	Reference	
In dogs that eat feral pig meat, frequency of	<1/year	2(22.2)	30(22)	Reference	0.96
consumption (*n* = 9 | 136)	1–6x/year	2(22.2)	41(30.1)	0.73 (0.1–5.49)	
	7–12x/year	1(11.1)	12(8.8)	1.25 (0.10–15.11)	
	>12x/year	4(44.4)	53(39)	1.13 (0.2–6.55)	
Other access to pig carcasses	Yes	7(38.9)	101(42.1)	0.88 (0.33–2.34)	0.79
(*n* = 18 | 240)	No	11(61.1)	139(57.9)	Reference	
*Exposure to other at-risk dogs*					
Multiple hunting dogs in the	Yes	17(85)	192(77.1)	1.65 (0.47–5.84)	0.66
household (*n* = 20 | 249)	No	3(15)	56(22.5)	Reference	
	Don't know	0(0)	1(0.4)	0.18 (0.01–5.44)^†^	
Mated to a dog that hunts	Yes	6(30)	80(32.2)	0.92 (0.34–2.5)	0.94
(*n* = 20 | 248)	No	13(65)	159(64.1)	Reference	
	Don't know	1(5)	9(3.6)	1.36 (0.16–11.57)	
Mated to a dog that eats feral pig	Yes	2(11.1)	50(20.7)	0.5 (0.11–2.27)	0.50
meat (*n* = 18 | 241)	No	14(77.8)	175(72.6)	Reference	
	Don't know	2(11.1)	16(6.6)	1.56 (0.33–7.49)	
Lives with dogs that eat feral pig	Yes	8(47)	110(45.6)	1.18 (0.43–3.25)	0.23
meat (*n* = 17 | 241)	No	8(47)	130(53.9)	Reference	
	Don't know	1(5.9)	1(0.4)	16.25 (0.93–284.40)	
Lives with a *B. suis* positive	Yes	7(41.2)	9(3.7)	16.25 (4.93–53.56)	<0.001
dog (*n* = 17 | 245)	No	9(52.9)	188(76.7)	Reference	
	Don't know	1(5.9)	48(19.6)	0.44 (0.05–3.52)	
Region (*n* = 20 | 254)	North	20(100)	197(77.6)	11.94 (0.71–200.40)^†^	0.002
	South	0(0)	57(22.4)	Reference	

*Questionnaires were partially or fully completed by 274 owners. Where incomplete data were recorded, the denominator used in the percentage calculation is indicated in parentheses after the variable name (n = positive | negative)*.

* *p-value based on omnibus test of model coefficients*.

† *OR (95%CI) calculated using the Haldane-Anscombe correction*.

**Table 5:** Unconditional associations between *Brucella suis* seropositivity and signs in subset of dogs that had clinical signs consistent with brucellosis in last week (*n* = 75).

**Clinical sign**	**Categories**	**Number (%) of**		**OR (95% CI)**	***p*** -value^*^
		**Positive dogs**	**Negative dogs**		
Total number of dogs with signs		6(8)	69(92)		
Lethargy (*n* = 5 | 68)	Yes	2(40)	35(51.5)	0.91 (0.12–6.88)	0.23
	No	2(40)	32(47)	Reference	
	Don't know	1(20)	1(1.5)	16.00 (0.71–361.72)	
Anorexia (*n* = 5 | 66)	Yes	2(40)	21(31.8)	1.36 (0.21–8.8)	0.82
	No	3(60)	43(65.2)	Reference	
	Don't know	0(0)	2(3)	0.40 (0.16–10.12)^†^	
Fever (*n* = 4 | 69)	Yes	1(25)	18(26.1)	0.8 (0.08–8.18)	0.61
	No	3(75)	43(62.3)	Reference	
	Don't know	0(0)	8(11.6)	1.37 (0.06–28.96)^†^	
Lameness (*n* = 4 | 67)	Yes	1(25)	35(52.2)	0.27 (0.03–2.71)	0.38
	No	3(75)	28(41.8)	Reference	
	Don't know	0(0)	4(6)	1.10 (0.05–25.16)^†^	
Joint signs (*n* = 4 | 66)	Yes	1(25)	25(37.9)	0.47 (0.05–4.75)	0.55
	No	3(75)	35(53)	Reference	
	Don't know	0(0)	6(9.1)	1.28 (0.06–27.85)^†^	
Back pain (*n* = 4 | 63)	Yes	1(25)	10(15.9)	1.5 (0.14–15.96)	0.56
	No	3(75)	45(71.4)	Reference	
	Don't know	0(0)	8(12.7)	1.31 (0.06–27.67)^†^	
Ocular signs (*n* = 4 | 63)	Yes	0(0)	5(7.9)	0.96 (0.04–20.32)^†^	0.44
	No	4(100)	51(81)	Reference	
	Don't know	0(0)	7(11.1)	01.31 (0.06–26.87)^†^	
Orchitis (*n* = 3 | 45)	Yes	1(33.3)	16(35.6)	0.88 (0.07–10.43)	0.93
	No	2(66.6)	28(62.2)	Reference	
	Don't know	0(0)	1(2.2)	0.26 (0.01–8.30)^†^	
Abortion history (*n* = 3 | 90)	Yes	1(33.3)	8(8.9)	9.75 (0.56–171.23)	0.11
	No	1(33.3)	78(86.7)	Reference	
	Don't know	1(33.3)	4(4.4)	19.50 (1.02–371.94)	

*Where incomplete data were recorded, the denominator used in the percentage calculation is indicated in parentheses after the variable name (n = positive | negative)*.

* *p-value based on omnibus test of model coefficients*.

† *OR (95%CI) calculated using the Haldane-Anscombe correction*.

Variables considered for inclusion in multivariable model analyses *(p* < 0.25) were sex, hunting frequency, hunting away, holding/lugging, household contact with a dog that eats feral pig meat, household contact with a *B. suis* positive dog, and region. The final model, which explained 26% of the variation in seropositivity, contained two statistically significant explanatory variables: hunting away and living with a *B. suis* positive dog (Table 6). The model predicted that dogs that hunted feral pigs away from their home regions and lived in households with seropositive dogs were four and 25 times, more likely to be seropositive than other dogs, respectively. The area under the ROC curve for this model was 0.78, indicating a satisfactory ability to predict *Brucella* serostatus in at-risk dogs.

**Table 6:** Estimated regression coefficients and their standard errors from a logistic regression model of risk factors for *Brucella suis* seropositivity in at-risk dogs.

**Variable**	**Coefficient (SE)**	**z**	***p*** -value^*^	**OR (95% CI)**
Intercept	−3.79(0.57)	−6.65	<0.001	-
Hunting away	1.37(0.64)	2.13	0.03	3.92 (1.12–13.74)^†^
Lives with a seropositive dog (Yes)	3.22(0.73)	4.39	<0.001	24.92 (5.94–104.59)
Lives with a seropositive dog (Don't know)	−0.78(1.08)	−0.72	0.47	0.46 (0.06 −3.80)

*The final model is based on 15 seropositive and 224 seronegative dogs*.

* *p-value based on backward stepwise (WALD)*.

† *Interpretation: The odds of being B. suis positive for dogs with a history of hunting away from their home region was 3.92 (95% CI 1.12 to 13.74) times that of dogs that did not hunt away. Area under the ROC curve = 0.78*.

## Discussion

This is the first comprehensive study to report the seroprevalence and risk factors for *B. suis* in at-risk dogs. Brucellosis due to *B. suis* biovar 1 has been recognized as an emerging disease in dogs not only in Australia but in countries such as the USA and Argentina (22, 55–57). Given its zoonotic potential, management of these dogs range from euthanasia to treatment regimens similar to those used for brucellosis in humans (4, 58–61). In this study one in 10 at-risk dogs were seropositive for *B. suis* with a higher seroprevalence detected in dogs from the northern half of the study region. After adjusting for likely confounders, hunting feral pigs away from their home location and sharing a household with a *B. suis* seropositive dog were associated with *B. suis* seropositivity. Clinical presentation was not associated with serostatus; seropositive dogs may or may not present with clinical signs. Conversely, seronegative dogs may present with signs consistent with brucellosis.

In a previous study in Hawaii, the apparent seroprevalence of *B. suis* in hunting dogs was found to be 5%. In that study, the feral pig population was estimated to have a seroprevalence of between 10 and 21% (56). We detected a similar apparent seroprevalence of 6.6% in at-risk dogs of eastern Australia, where the seroprevalence in feral pigs ranges between 0 and 17% (11). However, after adjusting for sampling effort and imperfect diagnostic test procedures, we found that the survey-adjusted true seroprevalence in at-risk dogs in eastern Australia was 9.3%. We believe this figure is a more accurate representation of the level of exposure in these dogs. The difference in the apparent and true seroprevalence values is attributed to the low combined sensitivity (0.45) of the RBT and CFT tests, which increases the likelihood of false negatives.

The sampling area in our study spanned a distance of approximately 900 kilometers, from Moonie QLD in the north to Bega NSW in the south. The risk of dogs testing positive varied according to location, with all seropositive cases located in the northern half of the study area. This correlates with the higher prevalence of *B. suis* in feral pigs observed in northern regions by Ridoutt et al. (11). As feral pigs are the reservoir for *B. suis* in Australia, their population density and distribution are likely to be important determinants of exposure in dogs. The Central and Northwest regions of NSW have previously recorded medium-sized feral pig populations with higher density pockets (62). Between 2009 and 2016 there was a southern expansion of the feral pig population into the Central West of NSW that coincided with the detection of *B. suis* infections in hunting dogs in NSW (21). The Central West, which recorded the highest seroprevalence in dogs in our study, is also home to major feral pig hunting events such as “Swine Time” (63), which attracts hunters to the area and confirms the presence of an active local hunting population. Although we did not detect any seropositive dogs in the southern most regions of our study, the movement of feral pigs from north to south (62) potentially increases the risk of spread and establishment of *B. suis* in southern feral pig populations.

Our study deliberately targeted dogs at-risk for *B. suis* through hunting and other practices, and therefore we did not assess the effect of hunting as a risk factor for *B. suis* seropositivity. Hunting was previously identified as the likely exposure route in the vast majority of dogs in Australia (21) and given this previous observation, our aim was to explore hunting and husbandry practices in more detail to better understand factors within this population that may increase risk. Surprisingly, the close contact hunting technique of holding and lugging, although employed by 100% of seropositive dogs, was not a significant risk factor for seropositivity when adjusted for other factors. Likewise, consumption of pig meat and access to the feral pig carcasses were not statistically significant risk factors even though consumption of meat infected with *B. suis* has been documented as causing clinical brucellosis in dogs in other studies (61, 64). Our findings are likely due to the lack of a comparative group, as all dogs in our study had exposure to feral pig blood and tissue, either through hunting or feeding practices.

Two significant risk factors were identified in our study: hunting outside or away from the dogs' home region and living with a *B. suis* seropositive dog. Of the dogs that hunt away from their home region, 73% (72 of 98) hunted in multiple locations, with distances ranging from a few kilometers into adjacent regions, to interstate travel over thousands of kilometers as shown in Figure 1. It has been recognized previously that hunters travel long distances to hunt and invest substantially in the activity of hunting (18, 19, 65). It would be reasonable to assume that hunting expeditions away from a home region would be to areas with large feral pig populations and that, due to the level of investment required for traveling these dogs may hunt more intensively, thereby engaging with and catching more pigs. Our questionnaire did not ask the owners to differentiate between the frequency of hunting and the success of hunting so this association could not be assessed. Dogs that travel for hunting are also more likely to be exposed to different pig populations which may increase their risk of exposure to pigs infected with brucellosis.

Our results showed that sharing a household with a seropositive dog was strongly associated with testing seropositive for brucellosis. All of the seropositive dogs within this group shared water, feed or sleeping areas with other dogs within the household. It is common for hunters to own multiple dogs (66); indeed 78% of our study dogs lived with other hunting dogs. Multiple seropositive dogs from the same household have been reported previously (21, 22, 27) and this finding highlights two possible transmission pathways for this population. The first can be explained by dogs being exposed to the same primary source at the same or similar time, namely the hunt, the meat or the equipment. The second possible pathway is within household (dog-to-dog) transmission. Much of the understanding of the pathobiology and epidemiology of *B. suis* in dogs has been extrapolated from *B. canis*, which also causes abortion, orchitis/epididymitis, discospondylitis, septic arthritis, meningoencephalitis, uveitis and lymphadenopathy in dogs (67–69). *Brucella canis* is highly transmissible. Shed readily in urine and reproductive fluids, it has also been isolated in blood, saliva, nasal and ocular secretions and feces (68). It has been postulated that *B. suis* may be spread the same way between dogs (70) however with the exception of reproductive fluids and tissue (26) there is little evidence at present that *B. suis* is shed in urine, saliva or feces. Consequently, the risk of transmission of infection between dogs may be considered minimal but further research is required to confirm and quantify the risk of dog-to-dog transmission.

Dogs in our study commonly presented with clinical signs such as lethargy, lameness/joint signs and orchitis, which could be attributable to brucellosis. Nonetheless, presence of clinical signs was not associated with serostatus. Since the first reported case of *B. suis* in a dog with fever and orchitis in 1931 (64), subsequent publications have identified a similar and expanding list of clinical presentations for this disease. Pyrexia, lethargy, epididymitis, back pain (23, 25, 71); abortions and vaginal discharge (72), discospondylitis (58); arthritis, abscesses, lymphadenomegaly (21); and pyothorax (55) have all been reported in confirmed *B. suis* cases. It is notable that none of these signs are specific to brucellosis and each has a differential diagnoses list of alternative causal organisms, conditions or injuries. The lack of a clear clinical picture or cluster of pathognomonic signs means that the history of exposure to feral pigs, directly or indirectly, becomes all the more important in the diagnosis and management of this disease. The potential for zoonotic transmission of *B. suis* to humans (26, 73, 74) highlights the need for appropriate diagnosis and treatment of these cases.

Diagnosing brucellosis can be difficult due to the lack of consistent clinical presentation, the cryptic and often latent nature of the infection and the reliance on imperfect serological tests (75). This is particularly problematic when life and death decisions are being made on the basis of such a diagnosis. Based on our study, a dog presenting with a history of feral pig-hunting from a household in southern QLD or NSW has a one in 10 chance of being seropositive for brucellosis. The probability of that dog being truly seropositive increases to an eight in 10 chance if both RBT and CFT tests are positive. The presence of clinical signs attributable to brucellosis would further increase the index of suspicion of true infection in such cases and may influence the decisions made around the management of these dogs. The current recommendation for seropositive dogs in Australia is treatment with doxycycline and rifampicin and desexing, or euthanasia (58, 76).

## Limitations

Although this study represents the most comprehensive investigation of *B. suis* in at-risk dogs to date, there are a number of limitations related to sample size, survey completion and sensitivity of the serology tests that should be mentioned. Despite a variety of approaches to recruitment, we were unable to achieve the target sample size in a number of regions. There are an estimated 200,000 to 300,000 recreational hunters in Australia (65) with approximately 100,000 to 200,000 engaged in feral pig hunting (18) and an estimated 52% of pig hunters using dogs for hunting (19). Hence the number of hunters in each region was not considered a limiting factor, rather their access and availability. Hunters come from across a wide demographic spectrum, both socio-economically and educationally, and hunt for a wide variety of reasons: income, land management, stock protection and recreation. As such there is a variation in the investment, attitude and professionalism toward both hunting and dog husbandry which can be reflected in the information obtained. Feral pig hunting can also be a contentious issue and some owners may wish to distance themselves and their dogs from the activity. The majority of the participants in this survey were enrolled through private veterinary clinics so the willingness of the dog owners to seek veterinary advice was likely a major factor, as was the relationship between the hunter and the veterinarian. The practice attitude to hunting may also play some role in the level of disclosure in the questionnaires. The laboratory tests currently available for *Brucella* spp. have low sensitivity and return an appreciable number of inconclusive results. The researchers are aware of more work being done in the area of *Brucella* testing in dogs at EMAI, with an ELISA and PCR test currently undergoing validation.

## Conclusion

This study reports for the first time a relatively high seroprevalence of *B. suis* in at-risk dogs in eastern Australia and identified hunting and husbandry practices associated with an increased risk of infection. It provides evidence of the geographical distribution of the disease from QLD, where the disease is endemic in feral pigs, to mid-central NSW. *Brucella* spp. are widely recognized as a cryptic organisms; causing infections that are difficult to diagnose accurately due to their often asymptomatic presentation and latent clinical behavior. Many questions remain unanswered about this emerging zoonotic disease and future studies are needed to define the progression and risk of transmission of *B. suis* infection in dogs, the merits of different serological and microbiological tests, the relevance of seropositivity in asymptomatic animals, the management of clinical cases and the efficacy of treatment protocols.

## Data Availability Statement

The data supporting the findings of this study are available from the corresponding author upon request.

## Ethics Statement

The study protocol was approved by the NSW Department of Primary Industries Animal Ethics Committee (AEC 2016/732), the University of Sydney Animal Ethics committee (AEC 2017/1278) and Human Research Ethics (HREC 2016/732) and the University of Melbourne Psychology Health and Applied Sciences Human Ethics Sub-Committee (ID 1748654). Written informed consent was obtained from the owners for the participation of their animals in this study.

## Author Contributions

CK coordinated data collection and testing, collected data, and conducted the data analysis and drafted the manuscript. KS and KW contributed to the study design and revised the manuscript. RM reviewed the literature and revised the manuscript. MS contributed to data analysis and drafted the manuscript. SM and AW designed the study, coordinated data collection and testing, supervised the project, and contributed to data analysis and drafted the manuscript. All authors read and approved the final manuscript.

## Funding

This research received funding from the New South Wales Department of Primary Industries Game Licensing Unit. CK was supported through a student scholarship from the University of Melbourne during the study period.

## Conflict of Interest

KS owns the company Kate Sawford Epidemiological Consulting Pty Ltd. The remaining authors declare that the research was conducted in the absence of any commercial or financial relationships that could be construed as a potential conflict of interest.

## Publisher's Note

All claims expressed in this article are solely those of the authors and do not necessarily represent those of their affiliated organizations, or those of the publisher, the editors and the reviewers. Any product that may be evaluated in this article, or claim that may be made by its manufacturer, is not guaranteed or endorsed by the publisher.
